# HGF Accelerates Wound Healing by Promoting the Dedifferentiation of Epidermal Cells through **β**_**1**_-Integrin/ILK Pathway

**DOI:** 10.1155/2013/470418

**Published:** 2014-01-15

**Authors:** Jin-Feng Li, Hai-Feng Duan, Chu-Tse Wu, Da-Jin Zhang, Youping Deng, Hong-Lei Yin, Bing Han, Hui-Cui Gong, Hong-Wei Wang, Yun-Liang Wang

**Affiliations:** ^1^The Neurology Department of the 148th Hospital, 20 Zhanbei Road, Zibo 255300, China; ^2^Department of Experimental Hematology, Beijing Institute of Radiation Medicine, 27 Taiping Road, Beijing 100850, China; ^3^Medical Research Center of Naval General Hospital, 6 Fucheng Road, Beijing 10037, China; ^4^Wuhan University of Science and Technology, Wuhan, Hubei 430081, China; ^5^Department of Medicine, The University of Chicago, Chicago, IL 60637, USA

## Abstract

Skin wound healing is a critical and complex biological process after trauma. This process is activated by signaling pathways of both epithelial and nonepithelial cells, which release a myriad of different cytokines and growth factors. Hepatocyte growth factor (HGF) is a cytokine known to play multiple roles during the various stages of wound healing. This study evaluated the benefits of HGF on reepithelialization during wound healing and investigated its mechanisms of action. Gross and histological results showed that HGF significantly accelerated reepithelialization in diabetic (DB) rats. HGF increased the expressions of the cell adhesion molecules *β*_1_-integrin and the cytoskeleton remodeling protein integrin-linked kinase (ILK) in epidermal cells *in vivo* and *in vitro*. Silencing of ILK gene expression by RNA interference reduced expression of *β*_1_-integrin, ILK, and c-met in epidermal cells, concomitantly decreasing the proliferation and migration ability of epidermal cells. *β*_1_-Integrin can be an important maker of poorly differentiated epidermal cells. Therefore, these data demonstrate that epidermal cells become poorly differentiated state and regained some characteristics of epidermal stem cells under the role of HGF after wound. Taken together, the results provide evidence that HGF can accelerate reepithelialization in skin wound healing by dedifferentiation of epidermal cells in a manner related to the *β*_1_-integrin/ILK pathway.

## 1. Introduction

Skin wound healing is a multifaceted process of reepithelialization that requires epidermal cell proliferation and migration, collagen fiber rearrangement, and cutaneous adnexa repair [1–3]. These epidermal cells are terminally-differentiated, but the molecular mechanisms involved in their proliferation and migration remain incompletely understood. According to Jones' report, if the differentiated epidermal cells high expressed *β*_1_-integrin, they would have a stronger ability to form clones and passage and generate a complete epithelium after moving them to skin wounds [4, 5]. Therefore, high expressed *β*_1_-integrins can prompt epidermal cells into a high proliferative and dedifferentiated state. It is well known that hepatocyte growth factor (HGF) regulates cell growth, cell motility, and morphogenesis in various types of cells, including epithelial and endothelial cells, supporting the hypothesis that it promotes epithelial repair and neovascularization during wound healing [6–9]. However, there are few reports that HGF was associated with *β*_1_-integrin in the process of promoting wound healing. In the present study, a plasmid carrying the HGF gene (PUDKH) was locally injected into the injured skin of diabetic rats, causing the plasmid-treated group to exhibit accelerated wound healing and increased expression of *β*_1_-integrin in epidermal lays and the molecular surface marker of epidermal stem cells. Overexpression of HGF in scratched primary rat epidermal cells also increased the expression of *β*_1_-integrin, confirming the result in a more isolated experimental system* in vitro*. *β*_1_-Integrin is thought to be one of specific markers for epidermal stem cells [10]. ILK was first discovered as a *β*_1_-integrin subunit binding protein. It localizes at the focal adhesions as well as sites of invasion and migration and is involved in cytoskeleton remodeling. Although the function of ILK has been intimately associated with integrin function, little association with HGF/c-met has been reported [11]. In this study, silencing ILK gene expression by RNA interference decreased the expressions of *β*_1_-integrin and c-met, concomitantly reducing the proliferation and migration ability of epidermal cells. These data demonstrate that HGF can accelerate skin wound healing by promoting the dedifferentiation of epidermal cells, while this process is closely related to ILK, an intracellular effector of cell-matrix interactions.

## 2. Materials and Methods

### 2.1. Reagents, Antibodies, and the Expression Vectors

Male Wistar rats were purchased from the Animal Center of the Academy of Military Medical Sciences (9-week old and weighing 270–300 g); Ham's F12 nutrient medium (Ham's F12), recombinant human HGF protein, and MTT were purchased from Gibco (Grand Island, New York, USA). Dimethyl sulfoxide and streptozotocin (STZ) were bought from Sigma (Austin, Texas, USA). All antibodies were obtained from R&D systems (Minneapolis, Minnesota, USA). The plasmid carrying human HGF cDNA was constructed by a colleague of the authors. The siRNAs against ILK and a nontarget siRNA were from Shanghai Gene Chemical Company (Songjiang, Shanghai, China).

### 2.2. Preparation of Animal Model [12]

The rate of wound healing in animal is usually more faster than that of human beings. So the effect of HGF will not be easy to be observed in the rapid wound healing process, while chronic wound healing is a troublesome and common complication of diabetes. Therefore rats were made diabetic by a single intraperitoneal injection of streptozotocin (STZ, Sigma Company) in this study. According to previous report, the diabetic rats actually showed significantly delayed wound healing than nondiabetic rats [13]. Rats with STZ-induced diabetes were fed a high fat diet (HFD) during the whole experimental period, whereas control rats were fed with basal diet (BD) served at the same time. HFD was prepared by adding sucrose (20%, w/w) and lard (20%, w/w) into BD. After 5 weeks, a single intraperitoneal injection of STZ (40 mg/kg dissolved in 100 mM citrate buffer pH 4.5) was administered to rats fed with HFD. Control rats received an equivalent volume of citrate buffer by intraperitoneal injection. Blood glucose levels were measured 72 h after STZ injection by tail vein puncture blood sampling using a hand-held glucometer (Changsha Sinocare Inc., China). Serum triglyceride (TG) and total cholesterol (TC) were determined by an autobiochemical analysis system (AU2700, Olympus, Japan), and body weight was recorded every week. Rats with blood sugar values at least 11.6 mmol/L were used for this study. During this period DB rats showed clinical signs of diabetes mellitus, for example, polyuria, polyphagia, and weight loss.

All DB rats were randomly divided into three groups (PUDKH group, PUDK group, and PBS group) (*N* = 20). DB rats were anesthetized for wounding with an intraperitoneal injection of sodium pentobarbital (0.5 mL/kg), and the hair on their back was clipped and the skin was cleaned. A round full-thickness wound measuring 2 cm in diameter was then made on the back of each animal using a 2 cm round scalpel. Wounds on rats in the PUDKH group were dressed with 50 *μ*g PUDKH per square centimeter by high-pressure syringe, while PUDK and PBS control rats were, respectively, treated with the same amount of empty plasmid and PBS. The wound areas of six DB rats were measured on 1, 3, 5, 7, and 14 d after gene transfer. Measurements were made with the aid of an image analyzer and a VEV image analysis software package (both from Di Meide Science and Technology Co., Ltd., Beijing). Two rats were killed at each time point, and skin samples were fixed in 4% formalin solution, embedded in paraffin, and sectioned (4 *μ*m) for histopathological evaluation.

### 2.3. Immunofluorescence and Immunohistochemistry

In the first stage of skin wound healing, inflammatory exudate and blood crust formation are dominant. The obvious reepithelialization phenomenon usually starts after 5 days. We therefore chose to detect the expressions of *β*_1_-integrin and ILK in rat skin tissue 7 d after gene transfer. Immunofluorescence (IF) staining was used to detect *β*_1_-integrin in epidermis during wound healing. Skin tissues were fixed in ice-cold methanol for 10 min and washed in PBS. After being blocked with 2% BSA in PBS, primary antibodies were applied overnight in a moist chamber set at 4°C and then rinsed with PBS three times for 5 min each rinse. Samples were then incubated with the 1 : 200 diluted tetraethyl rhodamine isothiocyanate- (TRITC-) conjugated-goat and anti-mouse immunoglobulin G secondary antibodies for 45 min in a dark incubation chamber at 37°C. After the skin tissues were washed in PBS, DAPI (1 : 1000) was used for nuclear staining. All specimens were examined under a fluorescence microscope (IX71-A12FL/PH, Olympus, Japan). Negative controls were prepared by incubation with the secondary antibody alone.

Immunohistochemistry was used to detect the expression of ILK. Four-micrometer paraffin sections were subjected to antigen retrieval using a pressure cooker, in sodium citrate (pH 6.0), for 4 minutes. Endogenous peroxidase was blocked with 3% hydrogen peroxide (H_2_O_2_) in PBS followed by nonspecific blocking with 2% PBS + bovine serum albumin (BSA) for 15 minutes. The sections were incubated with the primary antibody overnight at 4°C. After washing with PBS, slides treated with biotin-labeled secondary antibodies (1 : 500, R&D, USA) were incubated at RT for 1 h. The chromogenic reagent DAB was used to show the antibody conjugation. The intensity of the reaction observed on the slides was qualitatively analyzed.

### 2.4. Isolation and Culture of Epidermal Cells

Briefly, ultrathin epidermal sheets (grafted or ungrafted) were cut into pieces, digested with 0.25% trypsin for 20 min at 37°C, and made into single cell suspensions. After centrifugation, the epidermal cells were gently resuspended in Epilife medium supplemented with 1% human epidermal cells growth supplement and seeded on collagen IV coated culture flasks at a density of 5 × 10^5^ epidermal cells/cm^2^. After 24 hrs, nonadherent cells were gently removed.

### 2.5. *In Vitro *Studies Using Rat Epidermal Cells

For wounding experiments *in vitro*, epidermal cells were cultured to 100% confluency. The monolayer was scratched with a sterile needle to give a 0.8 mm wide wound, washed twice, and cultured in culture media with rat HGF (50 ng/mL). Inhibition of ILK expression in epidermal cells with ILK-specific siRNA reagents was performed as described previously [14]. Small interfering RNAs (siRNA) for rat ILK were synthesized by Shanghai Gene Chemical Company. siRNAs were transfected into the cells using Lipofectamine 2000 reagent (Invitrogen Life Technologies).

Images of wound areas were captured with Moticam (Motic Microscopes). Total scratch wound area was measured using Image Plus Software, and the percentage of wound closure at each time point was derived by following the formula: (1-[current wound size/initial wound size]) × 100 [15]. Following treatment, cells were washed in ice-cold PBS, and total cell lysates were prepared by scraping the cells in lysis buffer. Lysates were rotated at 4°C for 1 h and the insoluble material was removed by centrifugation at 12,000 ×g for 10 min. Equal amounts of denatured proteins were separated by 12% SDS-PAGE and transferred to PVDF membranes (Pharmarcia). The membranes were blocked by incubation in Tris-buffered saline nonfat dry milk for 2 h, followed by incubation at room temperature with indicated antibodies (against ILK, c-met, and *β*-Actin) at room temperature for 2 h. After extensively washing in Tris-buffered saline containing 0.1% Tween-20, the membranes were incubated for 1 h with horseradish peroxidase-conjugated secondary antibody. Membranes were then washed and developed using enhanced chemiluminescence substrate (ECL, Amersham Pharmacia Biotech).

After being scratched, the cells were cultured with HGF (50 ng/mL) for 48 h. The cells were then fixed in 4% paraformaldehyde in 0.1 M phosphate buffer (PBS, pH 7.4) for 15 min at room temperature and washed three times with phosphate buffered saline (PBS; 0.01 M phosphate, pH 7.3, 0.15 M NaCl). Next, cells were incubated in blocking buffer (PBS with 0.02% sodium azide, 0.2% Triton X-100, and 10% serum) for 1 h at room temperature. For immunoperoxidase labeling, cells were incubated in primary antibody (*β*_1_-integrin, CK19, and CK10) overnight at 4°C. After washing three times with PBS, cells were incubated in secondary biotinylated antibody for 2 h at room temperature. The chromogen was diaminobenzidine (DAB; 0.5 mg/mL in PBS) with 0.12% H_2_O_2_. After immunostaining, cells on coverslips were mounted and analyzed by an image analysis system. In the negative control, the antibodies were replaced with PBS.

### 2.6. Cell Migration Assay

Assessment of cell migration was performed as recently described [16] with minor modifications. Epidermal cells were dislodged after brief trypsinization and dispersed into homogeneous single cell suspensions that were washed extensively with DMEM/0.1% acid-free bovine serum albumin (migration medium) and resuspended in the same medium. Cells (1 × 10^5^) were dispersed onto collagen-coated chemotaxis filters that partition transwell inserts into upper and lower chambers. Migration medium (600 *μ*L) was placed in the lower chambers and the cells were allowed to adhere onto transwells for 1 h at 37°C. The medium in the lower chambers was then removed and cells were challenged by adding 600 *μ*L of fresh migration medium containing 0 or 50 ng/mL HGF into the lower chamber. Migration was allowed to proceed for 2 h at 37°C. Cells remaining attached to the upper surface of the filters were carefully removed with cotton swabs. The numbers of migrating cells in at least 10 consecutive fields were enumerated and their average was calculated after crystal violet staining. Data were expressed as the number of migrating cells per field.

### 2.7. Statistical Analysis

All data were expressed as mean ± standard deviation (X ± SD). Comparisons between groups were made using one-way analysis of variance. A *P* value of less than 0.05 was considered to be statistically significant. The results of immunofluorescence and immunocytochemistry were analyzed by an image analysis system. The integrated optical density (IOD) values of 10 fields were randomly determined in each sample under a microscope with the resulting IOD values used to do statistical analysis. The IOD and gray values were assayed by Image-Pro Plus 5.0 image analyzer (Media Cybernetics, USA).

## 3. Results

### 3.1. Wound Lesion Size and Histopathological Observation

**Figure 1:**
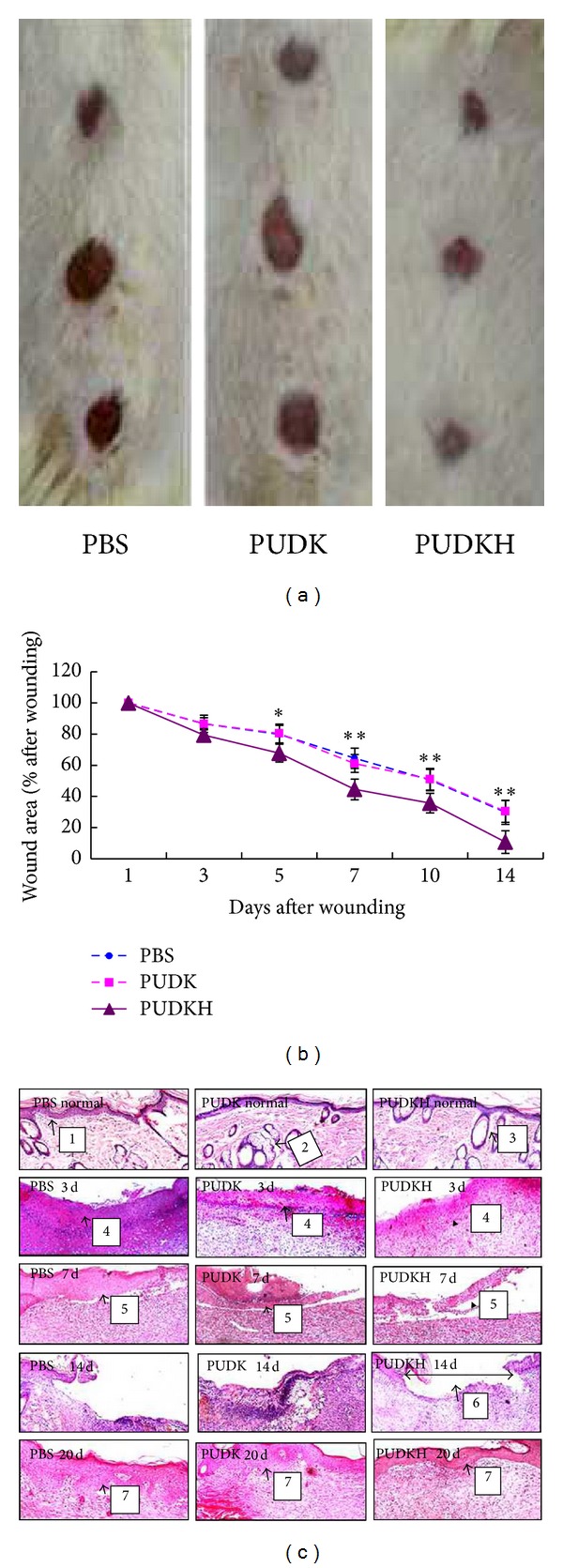
The gross and histological observation. (a) Photographs of full-thickness excisional punch wounds created in the skin of DB rats using a 2 cm biopsy tool 14 d prior to photography. After 14 d, the PUDKH group rat wounds are almost healed, while it took 20 days for wounds on rats in PBS and PUDK groups to heal. (b) Photographs of wounds were captured on 1, 3, 5, 7, 10, and 14 days after wounding to determine the degree of wound closure in DB rats. Graph represents the percentage of wound area at different times after wounding. The *P* value means that there are differences between PUDKH group versus the PBS and PUDK groups animals at matched time point (**P < 0.01). (c) Hematoxylin/eosin staining showed a regularly stratified epithelium with ordinary developed hair follicles in normal rats. Reepithelialization of wounds in HGF gene-transfer rats was significantly increased on 5, 7, 10, and 14 days than that of the PBS and PUDK groups after gene transfer (*P < 0.05). On 14 d, regenerated epidermal cells layers recovered skin wounds of DB rats in PUDKH group; until 20 days, the rats in PBS and PUDK groups wound healed. 1: The epidermis of normal rat skin, 2: the sebaceous gland of normal rat skin, 3: the hair follicle of normal rat skin, 4: granuloma in rat skin wounds on 3 d, 5: reepithelialization in the wound edges on 7 d, 6: the regenerated epidermal cells layers recovered skin wounds on 14 d in PUDKH group, and 7: the newly-formed epithelium on 20 d.

Gross observation of dorsal wound revealed that a reduction of the wound area in the PUDKH group was qualitatively visible (Figure 1(a)). The wound areas of six DB rats in PBS, PUDK, and PUDKH groups were measured at 1, 3, 5, 7, and 14 d after HGF gene transfer (specific wound area values are not shown). Measurements were made with the aid of an image analyzer and an image analysis software package. As shown in Figure 1(b), it is obvious that the degree of reepithelialization of the wounds in HGF gene-transfer rats (PUDKH group) was significantly increased after 5, 7, 10, and 14 days compared to the PBS and PUDK groups following gene transfer (*P < 0.05). At 14 d, the PUDKH group rat wounds were almost healed, while it was 20 days until the wounds in the PBS and PUDK rats had healed.

In Figure 1(c), tissue sections from normal rats (hematoxylin/eosin staining) showed a regularly-stratified epithelium with ordinary developed hair follicles. In experimental animals, the first stage of skin wound healing is dedicated to hemostasis and the formation of a provisional wound matrix, initiating the inflammatory process. Next, neovascularization and angiogenesis are activated, marked by the immigration of local fibroblasts along the fibrin network and the beginning of reepithelialization of the wound edges. In the process of wound healing, rapid reepithelialization may prevent pathological scar formation to some extent. Interestingly, the newly-formed epithelium in PBS and PUDK groups was very thick, with more epidermis nipple in the base and coarse/disordered collagen fibers in the dermal layer. This seems to confirm the above view. These results indicated that HGF gene transfer into skin wound may have aided reepithelialization in wound healing. To further explore the specific mechanisms, we measured *β*_1_-integrin and ILK expression in DB rats wounds.

### 3.2. HGF Promotes the Regeneration of Epidermal Cells Expressing *β*_1_-Integrin and ILK

**Figure 2:**
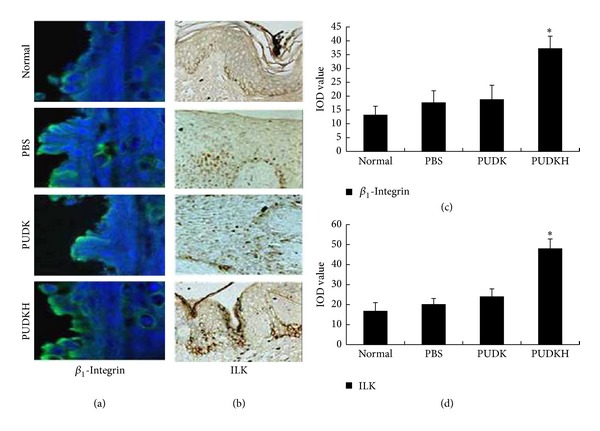
HGF promotes *β*_1_-integrin and ILK expression in rat epidermal cells. (a) Rat skin tissues transfected with HGF gene on the PUDK plasmid (PUDKH group) and control rats (empty vector (PUDK) group and vehicle (PBS) group) skin tissues were fixed and stained with anti-*β*_1_-integrin antibody and fluorescein isothiocyanate (FITC) conjugated secondary antibody (green) and nuclei visualized with Hoechst 33342 (blue). (c) A representative image is shown. All four groups expressed blue fluorescent protein in epidermal prickle cell or in the granule cell layer in the process of wound regeneration. But, after analysis by Image-Pro Plus 5.0 image analyzer, we found that blue fluorescent proteins expression was greatest in PUDKH group, yielding a statistically significant difference (*P < 0.05). (b) ILK expression and activity were evaluated in the injured skin treated with HGF gene transfection by immunohistochemical staining and compared to healthy skin from the control animals. ILK expression was observed in healthy skin and was especially abundant in the basal epidermis. After injury, an increase in ILK staining was observed in the wounded area of PUDKH group than in PBS, PUDK, and normal group. (d) A representative image is shown. The results of immunohistochemistry were analysed by Image-Pro Plus 5.0 image analyzer; we found that IOD value was greatest in PUDKH group than in the PBS, PUDK, and normal group, yielding a statistically significant difference (*P < 0.05).

Frozen sections of skin epidermis from the three groups were made 7 d after gene-transfer. The expression of *β*_1_-integrin, the epidermal stem cell molecular surface marker, was detected by immunofluorescence staining. As shown in Figure 2(a), all the four groups expressed blue fluorescent protein in epidermal prickle cell or in the granule cell layer during wound healing. Quantitative analysis using Image-Pro Plus 5.0 image analyzer revealed that blue fluorescent protein expression was significantly higher in PUDKH group than that of the three groups (*P < 0.05) (Figure 2(c)). This indicated that *β*_1_-integrin in epidermis on the edge of the round wound was significantly increased in HGF gene-transfer rats 7 days after gene transfer, compared with those in control rats. After 7 days, ILK expression and activity were evaluated in the injured skin by immunohistochemical staining and compared to healthy skin from the control animals. ILK expression was observed in healthy skin and was especially abundant in the basal epidermis. After injury, an increase in ILK staining was observed in the wounded area of the PUDKH group compared to the PBS, PUDK, and normal groups (Figures 2(b) and 2(d)).

### 3.3. *In Vitro *Studies Using Primary Rat Epidermal Cells

**Figure 3:**
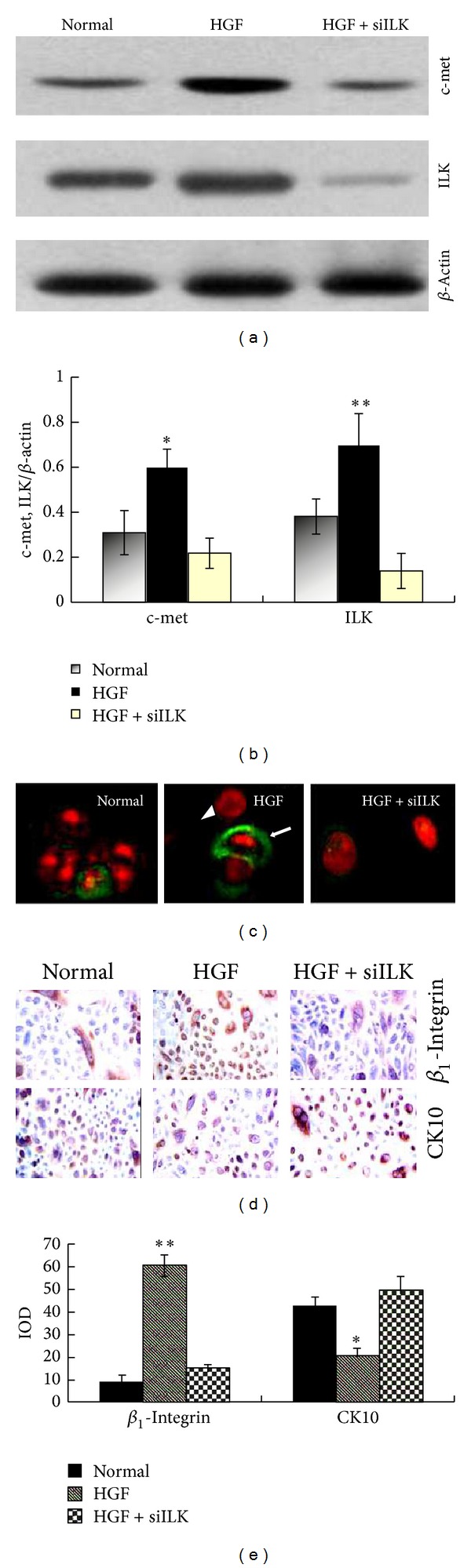
HGF promotes ILK and c-met expression in scratched epidermal cells. (a) ILK and c-met expression in cultured epidermal cells was analyzed by western blot 48 h after scratch wounding. An increase of ILK and c-met expression was observed in epidermal cells treated with HGF (50 ng/mL) for 48 h. After epidermal cells were transfected with ILK siRNA, the expression of ILK and c-met was downregulated, despite the presence of HGF protein (50 ng/mL). (b) Graph represents densitometric analysis of western blots described in (a) (*P < 0.05,**P < 0.01). (c) The distribution of c-met, the receptor of HGF, was detected by IF. The c-met protein at the surface of the epidermal cells is green (white arrow) and in the nuclei is red (white triangle). (d) *β*_1_-Integrin and CK10 expression in scratched epidermal cells were tested by immunocytochemistry. HGF increased the expression of *β*_1_-integrin and downregulated CK10. However, ILK siRNA-transfected cells showed a significant knockdown of *β*_1_-integrin expression, with an increase in CK10 expression, compared to nontransfected or control cells. (e) Graph represents densitometric analysis of western blot described for (d) (*P < 0.05, **P < 0.01). The results revealed that there was a statistically significant difference between HGF group versus normal and HGF + siILK groups. All these suggested that HGF could activate the *β*_1_-integrin and ILK signaling pathway; inversely, *β*_1_-integrin and ILK might regulate the expression and function of HGF/c-met.

The possible role of HGF in accelerating the *β*_1_-integrin and ILK expression was assayed by RNA interference *in vitro*. Primary rat epidermal cells were isolated from newborn rat by dispase/trypsin treatment and cultured as previously described [17]. Once 100% confluent, cells were scratched with a sterile needle and HGF protein (50 ng/mL) was added to the conditions medium for 48 h. As an alternative method to deplete ILK expression, small interference RNA (siRNA) knockdown experiments targeting ILK were also performed. After isolating the total cell lysates, western blotting was used to semiquantitatively analyze the ILK and c-met expression. An increase of ILK and c-met protein was observed in epidermal cells treated with HGF (50 ng/mL) for 48 h, while, after ILK siRNA, the expression of ILK and c-met in epidermal cells was downregulated despite the presence of HGF (*P < 0.05, **P < 0.01). And the expression of c-met (the receptor of HGF [16]) was also detected by immunofluorescence; results were similar with the above conclusion. *β*_1_-Integrin and CK10 in scratched epidermal cells were measured by immunocytochemistry. Graph that represents densitometric analysis showed that HGF increased the expression of *β*_1_-integrin and downregulated CK10 (*P < 0.05, **P < 0.01). In summary, HGF increased the expressions of c-met, ILK, and *β*_1_-integrin but lowered the expression of CK10. However, ILK siRNA-transfected cells showed a significant decrease in ILK and *β*_1_-integrin expression, with no increase in c-met expression observed after wounding, compared to nontransfected or control cells (Figure 3). All these suggested that HGF could activate the *β*_1_-integrin and ILK signaling pathway. Inversely, the *β*_1_-integrin/ILK pathways might regulate the expression and function of HGF/c-met. In next study, we used wound healing and cell migration assay to examine that whether HGF functions were affected after ILK gene depletion.

### 3.4. ILK Depletion Inhibits Epidermal Cells Proliferation and Migration

**Figure 4:**
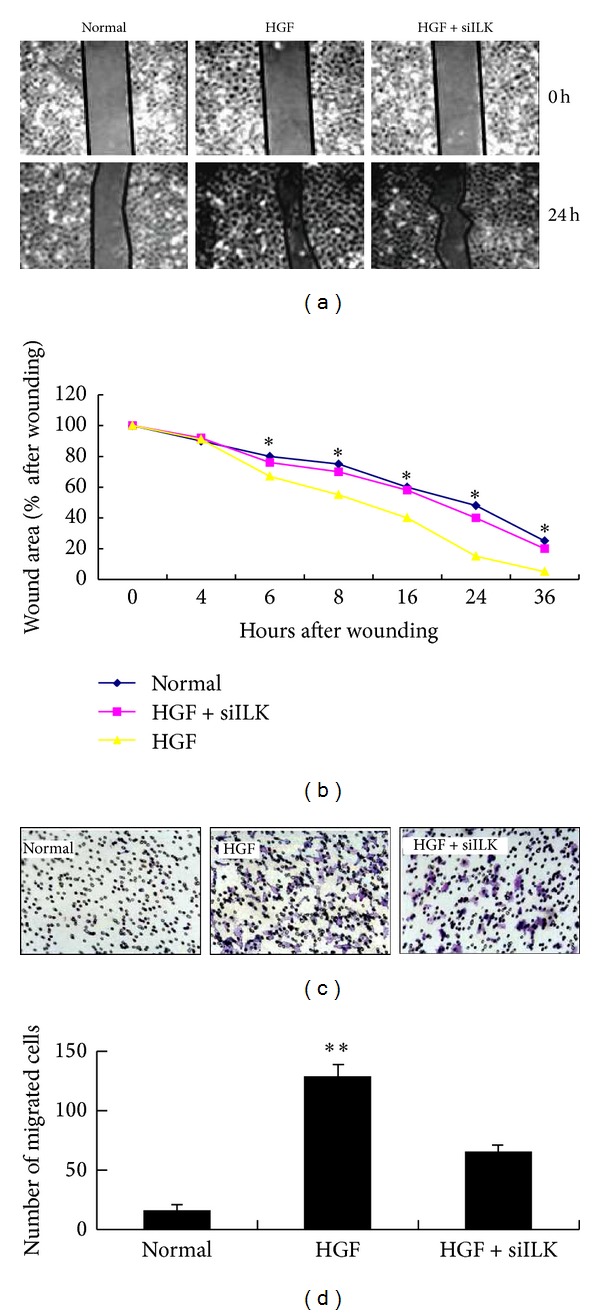
ILK knockdown epidermal cells, cell migration and invasion. (a) Epidermal cells treated with HGF (50 ng/mL) and ILK knockdown were grown to confluence and a wound was created. Photographs of wounds were captured at 0 or 36 h after wounding to determine the degree of wound closure. A representative experiment is shown. (b) Graphs represent the percentage of *t* = 0 h wound area at different times after wounding (*P < 0.05 HGF group versus normal and HGF + siILK groups at matched time point). (c) ILK knockdown epidermal cells were seeded onto migration chambers in triplicate and were allowed to migrate for 2 hours. Cells that migrated through the membrane were fixed and visualized by crystal violet staining. (d) Graphs represent the amount of cells in normal, HGF, and HGF + siILK groups. After ILK knockdown, the migration and invasion ability of epidermal cells were obviously declined (**P < 0.01 was regarded as statistically significant between HGF group versus normal and HGF + siILK groups).

To investigate whether the observed defects in wound closure were due to deficiencies in cellular migration or proliferation, scratch wound assays were repeated. The migratory ability of ILK knockdown epidermal cells during wound healing was measured using transwell migration chambers. Confluent cell monolayer wound healing requires cell migration from the leading edge and cellular proliferation to replace lost cells. Results showed differences between monolayer closure in the presence or absence of HGF protein and between epidermal cells treated with or without ILK siRNA-transfection (Figures 4(a) and 4(b)), indicating that the retarded closure is due to reduced proliferation of ILK-deficient cells. Cell invasiveness of ILK knockdown epidermal cells was assessed by seeding the cells onto matrigel-coated invasion chamber. In presence of HGF protein, more invaded cells were observed in invasion chamber, while significantly fewer cells were able to invade through the matrigel in ILK knockdown epidermal cells (Figures 4(c) and 4(d)). Taken together, these data demonstrate that ILK expression is necessary for HGF induction during wound healing* in vitro*.

## 4. Discussion 

Skin is the biggest organ of the human being and has many functions, including covering the whole body and protecting other tissues and organs. Therefore, the healing of a skin wound involves an extraordinary mechanism of cascading cellular functions that is unique in nature [1, 2, 6]. The process requires the regulation of a variety of cell types and growth factors, including epidermal cells, stromal cells, epidermal growth factor (EGF), fibroblast growth factor (FGF), and HGF, each playing a critical role by mediating reepithelialization, epidermal cell differentiation, fibrosis, and angiogenesis [6]. HGF is a multifunctional mediator of these processes. The half-life of exogenous HGF protein is relatively short, and even repeated infusions of HGF fail to maintain high levels of HGF *in vivo* [7]. To better understand the effects of HGF on wound healing, the plasmids carrying the HGF gene (PUDKH) were locally injected into the trauma skin of diabetic rat. The degree of reepithelialization of wounds in HGF gene-transfer rats was significantly increased on 5, 7, 10, and 14 days than that of PBS and PUDK groups.

During wound-induced cell proliferation, the main focus of the healing process is to recover the wound surface. The proliferation and migration of epidermal cells are crucial for the rapid closure of the epidermis [1, 2]. The reepithelialization process is mediated by local epidermal cells at the wound edges and by epithelial stem cells from hair follicles or sweat glands [18–21], while, according to previous report, if the differentiated epidermal cells high expressed *β*_1_-integrin, they would have a stronger ability to form clones and passage and generate a complete epithelium after moving them to skin wounds [4, 5]. Therefore, high expressed *β*_1_-integrins can prompt epidermal cells into a high proliferative and dedifferentiated state. In subsequent investigations, the model of aged epidermal cell dedifferentiation *in vivo* was constructed [22] and the dedifferentiation-derived stem cell-like cells were isolated [9].

In this study, we also found a significant increase in epidermal *β*_1_-integrin and ILK expressions on the edge of the round wound in HGF gene-transfer rats 7 d after gene transfer relative to control rats. *β*_1_-Integrins are essential for tissue development and maintenance [1, 21–26]. They are highly expressed on stem and progenitor cell populations, which orchestrate organogenesis and represent a cellular reservoir to maintain organ homeostasis [26, 27]. ILK, an integrin *β*_1_-subunit binding protein, is an intracellular effector of cell-matrix interactions and regulates many cellular processes, including growth, proliferation, survival, differentiation, migration, invasion, and angiogenesis [7, 28, 29]. We believe that HGF promoted the transformation of epidermal cells into dedifferentiated stem-like cells, characteristic of the powerful ability of proliferation and migration. In fact, epidermal cell proliferation and migration are very important in facilitating epithelial wound repair. HGF appeared to be involved in the dedifferentiation of epidermal cells, accelerating wound healing in a *β*_1_-integrin/ILK signaling pathway-related manner. Experiments *in vitro* verified these findings. The scratched epidermal cells showed ectopic expression of *β*_1_-integrin and ILK under the role of HGF. However, under the same conditions, as in 50 ng/mL HGF, the expression of *β*_1_-integrin and ILK was downregulated after ILK gene silencing by RNA interference, and c-met, the receptor of HGF, decreased within the epidermal cells plasma membrane. At the same time, wound healing and cell migration assays showed that the proliferation and migration ability of the epidermal cells were partly suppressed after ILK gene silencing. It must be noted that ILK deficiency leads to retarded wound closure in skin, while it is related to HGF expression after knockdown of ILK in Isabel Serrano's research report. And after exogenous administration of human HGF, alterations in cell proliferation and wound closure in ILK-deficient mouse embryonic fibroblast or mice could be observed [28, 29]. However, in our study, HGF can promote the expression of ILK in the process of wound healing, and the capacity of HGF to promote epidermal cell proliferation and migration will be affected after blocking ILK expression.

Our data demonstrate that HGF can accelerate wound healing by promoting the dedifferentiation of epidermal cells in a process closely related to the *β*_1_-integrin and ILK signaling pathways. Notably, dedifferentiation is observed in a variety of processes such as cancer, organ regeneration, and stem cell renewal, but it has been difficult to study because there are few experimentally tractable systems to identify it. Particularly in skin, dedifferentiation of the epidermal cells is still a matter of some debate [8, 9]. Although the finding of the differentiated cell dedifferentiation was just preliminary, it was confirmed that HGF increased the expression of *β*_1_-integrin in epidermal cells. Most importantly, HGF improved the proliferation and migration of epidermal cells. It is therefore possible that dedifferentiation-derived cells will be another source of epidermal stem cells for wound repair and regeneration in the future. Simultaneously, the potential powerful ability of HGF to promote skin wound healing should attract more attention.
